# Sarcopenia in Chronic Obstructive Pulmonary Disease: Prevalence and Association With Disease Severity in an Indian Tertiary Care Cohort

**DOI:** 10.7759/cureus.116691

**Published:** 2026-09-21

**Authors:** Aditya Gupta, Sunita Gupta, Manan Gupta

**Affiliations:** 1 General Medicine, Maharishi Markandeshwar University of Medical Sciences and Research, Ambala, IND

**Keywords:** chronic obstructive pulmonary disease, ewgsop2, gait speed, gold classification, handgrip strength, india, muscle mass, prevalence, sarcopenia

## Abstract

Background

Chronic obstructive pulmonary disease (COPD) has systemic effects beyond the lung, of which skeletal muscle deterioration is among the most consequential. Sarcopenia, defined by concurrent reductions in muscle mass, strength and physical performance, worsens disability and long-term outcomes. Indian data are sparse. The primary objective was to estimate sarcopenia prevalence among spirometry-confirmed, stable COPD patients at a tertiary care centre in northern India using European Working Group on Sarcopenia in Older People 2019 (EWGSOP2) criteria. The second pre-specified objective was to examine its association with COPD severity by Global Initiative for Chronic Obstructive Lung Disease (GOLD) stage. Exploratory analyses examined associations with sex, age group and body mass index (BMI).

Methods

This prospective cross-sectional study was conducted at Maharishi Markandeshwar Institute of Medical Sciences and Research, Mullana, Ambala, between March 2024 and June 2025. One hundred consecutive spirometry-confirmed COPD patients were enrolled. Sarcopenia was assessed using the three-component EWGSOP2 algorithm: a skeletal muscle index derived from bioelectrical impedance analysis (OMRON HBF-222T, Omron Healthcare, Kyoto, Japan) and applied against the EWGSOP2 appendicular skeletal muscle mass thresholds, handgrip strength by digital hand dynamometry, and gait speed obtained from the six-minute walk test. Sex-specific EWGSOP2 cut-offs were applied. BMI was classified using World Health Organization international cut-off points. Associations were tested by Pearson chi-square, with Fisher’s exact test where expected cell counts fell below five. Prevalence is reported with 95% Wilson confidence intervals (CIs).

Results

The cohort comprised 67 men and 33 women with a mean age of 56.69 years; 83 patients (83%) had severe or very severe airflow obstruction. Sarcopenia prevalence was 31% (95% CI 22.8-40.6), higher in men (40.3%) than women (12.1%; χ^2^ = 8.21, df = 1, p = 0.004). Sarcopenia was absent in the three patients with mild disease, present in five of 14 with moderate disease (35.7%) and in 26 of 83 with severe-to-very-severe disease (31.3%); this distribution was not statistically significant (χ^2^ = 1.50, df = 2, p = 0.473; Fisher’s exact p = 0.632). Sarcopenic patients had lower gait speed (men 0.61 m/s, women 0.60 m/s), handgrip strength (21.87 kg, 14.53 kg) and muscle index (6.47, 4.97) than non-sarcopenic patients. Mean BMI was lower in sarcopenic patients (19.58 versus 24.04 kg/m^2^, p < 0.001), and sarcopenia was associated with BMI category (χ^2^ = 26.82, df = 3, p < 0.001), affecting 73.7% of underweight and 30.2% of normal-weight patients and no overweight patient.

Conclusions

Sarcopenia affected nearly one-third of this Indian tertiary care COPD cohort and was more frequent in men and in those with low BMI. Prevalence was numerically higher in moderate and advanced disease than in mild disease, but no significant gradient across GOLD strata could be demonstrated in a sample in which 83% of patients had severe or very severe obstruction; this reflects the narrow severity distribution rather than evidence against a relationship. The cross-sectional design does not permit any causal interpretation of the observed associations. Routine assessment of muscle mass, strength and performance is feasible in resource-limited settings. Adequately powered multicentre studies using Asian-specific criteria are needed.

## Introduction

Chronic obstructive pulmonary disease (COPD) represents one of the foremost contributors to global morbidity and premature mortality, with the Global Burden of Disease consortium estimating more than three million attributable deaths annually [1]. COPD is fundamentally a disease of persisting airflow limitation accompanied by chronic respiratory symptoms; however, the pathological process is far from confined to the lungs [2]. Cardiovascular disease, metabolic disturbance, osteoporosis, and progressive skeletal muscle dysfunction together frame COPD as a multi-organ disorder whose burden is inadequately captured by spirometric indices alone [3].

Among these systemic complications, skeletal muscle wasting is of particular clinical consequence, and sarcopenia occupies a distinct position within it. In COPD, sarcopenia is regarded as predominantly secondary in nature, arising from the convergence of chronic systemic inflammation, sustained hypoxaemia, deconditioning driven by dyspnoea-related activity avoidance, intermittent corticosteroid exposure, and adverse hormonal and nutritional change [4]. Affected individuals show impaired exercise tolerance, more severe symptoms, greater exacerbation frequency, and higher mortality than COPD patients without sarcopenia [5]. Pooled estimates from recent systematic reviews place prevalence in the 21-29% range, with individual study estimates spanning below 10% in community samples to above 50% in inpatient and rehabilitation settings, reflecting the influence of study design, population characteristics, and diagnostic criteria [6].

For India, the landscape is substantially different. COPD prevalence is estimated at approximately 7% nationally but reaches considerably higher levels in occupational and biomass-exposed subgroups, and the disease tends to manifest at an earlier age than in developed countries owing to the dual burden of tobacco and household solid fuel exposure [7]. Despite this, published data on sarcopenia prevalence specifically within Indian COPD populations remain exceptionally limited.

The present study was therefore designed with two pre-specified objectives. The primary objective was to estimate the prevalence of sarcopenia, defined by EWGSOP2 criteria, among spirometry-confirmed, stable COPD patients attending a tertiary care teaching hospital in northern India. The second pre-specified objective was to examine the association between sarcopenia status and COPD severity as classified by the GOLD system. The further analyses are reported here. The associations of sarcopenia with patient sex, age group, and body mass index (BMI) category were not pre-specified objectives of the study and are therefore reported as exploratory analyses; they are presented to characterise the demographic and clinical phenotype of sarcopenic COPD patients in this population and should be interpreted as hypothesis-generating rather than confirmatory. Consistent with a cross-sectional design analysed by categorical methods, all these relationships are described throughout as associations rather than correlations.

## Materials and methods

Study design and setting

This prospective cross-sectional observational study was conducted at the Department of General Medicine, Maharishi Markandeshwar Institute of Medical Sciences and Research (MMIMSR), Mullana, Ambala, Haryana, a rural tertiary care teaching institution in northern India. Patient enrolment was carried out prospectively between March 2024 and June 2025. Institutional Ethics Committee clearance was obtained prior to commencement of the study (Project No. IEC-2871, approved 20 March 2024), and written informed consent was secured from each participant.

Inclusion and exclusion criteria

Eligible patients were screened according to predefined inclusion and exclusion criteria, summarised in Table 1. Consecutive patients attending the inpatient and outpatient services during the study period were screened for eligibility, and enrolment continued until the target of 100 patients had been reached. The number of patients assessed for eligibility and the reasons for exclusion were not recorded, so a screening flow diagram cannot be presented; this is acknowledged in the Limitations as a constraint on the assessment of selection bias. Patients presenting in acute exacerbation were excluded, so the cohort comprises patients assessed in the stable state.

**Table 1:** Inclusion and exclusion criteria COPD: chronic obstructive pulmonary disease; GOLD: Global Initiative for Chronic Obstructive Lung Disease; FEV₁: forced expiratory volume in one second; FVC: forced vital capacity.

Inclusion Criteria	Exclusion Criteria
Adults with spirometry-confirmed COPD (post-bronchodilator FEV₁/FVC <0.70) meeting GOLD diagnostic criteria	Acute exacerbation of COPD
Provision of informed consent	Pneumonia or septicaemia
Attending MMIMSR inpatient or outpatient services during the study period	Hypertension, type 2 diabetes mellitus, chronic kidney disease, chronic liver disease, or malignancy
	Any disorder affecting muscle mass, strength, or physical performance independently of COPD

Sample size estimation

A formal a priori sample size calculation was not performed. The target of 100 patients was set on the basis of the expected number of eligible COPD patients presenting to the department during the enrolment window. The precision achieved by that sample can nonetheless be stated: using the standard formula for estimation of a single proportion,

*n = Z2 &times; p(1 &minus; p) / d2*

where Z = 1.96 and an anticipated prevalence of p = 21.6% (the pooled estimate in the study by Benz et al. [6]), a sample of 100 corresponds to an absolute precision of approximately ±8% at 95% confidence. No claim is made that this precision is adequate for any particular purpose; it is reported so that readers may judge the resolution of the prevalence estimate for themselves. The sample was not powered to detect differences in prevalence across GOLD severity strata, and the severity analysis is accordingly underpowered.

Spirometry and COPD severity classification

All enrolled participants underwent post-bronchodilator spirometry according to standard recommendations. COPD severity was classified using the GOLD criteria [2] based on percentage predicted forced expiratory volume in one second (FEV₁): GOLD 1 (mild, ≥80%), GOLD 2 (moderate, 50-79%), GOLD 3 (severe, 30-49%), and GOLD 4 (very severe, <30%). Owing to the limited number of patients in the extreme severity categories, GOLD 3 and GOLD 4 were combined for statistical analyses, resulting in three severity groups: mild, moderate, and severe-to-very-severe COPD.

Sarcopenia assessment

Sarcopenia was assessed according to the 2019 EWGSOP2 recommendations [8], incorporating measurement of physical performance, muscle strength, and muscle mass as summarised in Table 2. Sarcopenia was confirmed when low muscle mass was present in conjunction with impaired physical performance and/or reduced muscle strength according to EWGSOP2 criteria. All three assessments were performed at a single study visit. The order in which they were performed, and the number of observers involved, were not recorded; this is acknowledged in the Limitations.

**Table 2:** EWGSOP2 criteria used for sarcopenia assessment EWGSOP2: European Working Group on Sarcopenia in Older People 2; 6MWT: six-minute walk test. The muscle-mass and physical-performance measures used here are not identical to those specified by EWGSOP2: muscle mass was assessed by a bipedal impedance device that does not report appendicular skeletal muscle mass directly, and gait speed was obtained from a six-minute walk rather than a four-metre usual-pace walk. Both deviations are detailed in the Methods and Limitations.

Parameter	Assessment Method	Cut-off Value
Muscle mass	Skeletal muscle index from bioelectrical impedance analysis	Men: <7.0 kg/m²; Women: <5.5 kg/m²
Muscle strength	Handgrip dynamometry	Men: <27 kg; Women: <16 kg
Physical performance	Gait speed obtained from 6MWT (deviation from EWGSOP2 4-metre walk)	≤0.8 m/s

Muscle mass: bioelectrical impedance analysis

Body composition was assessed using an OMRON HBF-222T body composition monitor (Omron Healthcare, Kyoto, Japan), acquired by the investigators in November 2023 and used for all participants throughout the study. This is a bipedal (foot-to-foot) device with four-foot electrodes, which passes a single-frequency 50 kHz current and, according to the manufacturer’s specification, reports body weight, body fat percentage, visceral fat level, skeletal muscle percentage, resting metabolism and BMI. The device does not perform segmental analysis and does not itself report appendicular skeletal muscle mass. The muscle-mass variable recorded in this study was a skeletal muscle index, which was compared against the EWGSOP2 appendicular skeletal muscle mass thresholds of 7.0 kg/m^2^ for men and 5.5 kg/m^2^ for women.

The manner in which the index was derived from the device output was not documented in the study records, and the proprietary nature of the manufacturer’s estimating algorithms is not disclosed. Both points are acknowledged in the Limitations as substantial constraints on comparability with studies using dual-energy X-ray absorptiometry or research-grade multi-frequency impedance systems. The manufacturer also notes that the device is intended for home use rather than professional use, that measurements may be inaccurate in the presence of oedema, and that it should not be used in patients with implanted cardiac devices; patients with clinically evident peripheral oedema and those with such devices were therefore not assessed.

Muscle strength: handgrip dynamometry

Muscle strength was measured using a Camry EH101 electronic hand dynamometer (Zhongshan Camry Electronic Co., Ltd., Guangdong, China), a digital instrument employing a strain-gauge sensor, with a measuring range of 0-90 kg, a resolution of 0.1 kg and a manufacturer-stated tolerance of ±0.5 kg. The handle was adjusted to the participant’s hand size before testing, and the device automatically captures and displays the maximum value achieved during each effort. Three attempts were performed with the dominant hand and the highest value was recorded. The participant’s position during testing and the rest interval between attempts were not recorded, and are acknowledged in the Limitations.

Physical performance: gait speed

Physical performance was assessed using gait speed obtained from the six-minute walk test (6MWT), performed according to American Thoracic Society/European Respiratory Society guidelines [9], and the EWGSOP2 threshold of ≤0.8 m/s was applied. The arithmetic by which gait speed was derived from the walk test was not documented, and the walk distances themselves were not retained; this is acknowledged in the Limitations. The length of the walking course, and whether supplemental oxygen was permitted during testing, were also not recorded. It must be emphasised that this measure is not equivalent to the EWGSOP2 physical performance criterion. EWGSOP2 specifies a four-metre usual-pace walk, whereas a six-minute average incorporates a sustained endurance component, turns, and the effects of exertional dyspnoea, and may therefore yield systematically different values. The 6MWT was used because it was already integral to the functional assessment of this cohort, and its use represents a deviation from the standard algorithm rather than an alternative implementation of it.

Anthropometry and BMI classification

BMI was calculated as weight in kilograms divided by height in metres squared (kg/m²) and classified according to the WHO international cut-off points [10]: underweight (<18.5 kg/m²), normal weight (18.5-24.9 kg/m²), overweight (25.0-29.9 kg/m²), and obese (≥30.0 kg/m²). The WHO international classification, rather than the lower Asian-specific action points proposed by the WHO Expert Consultation for Asian populations (overweight ≥23.0 kg/m²; obesity ≥27.5 kg/m²) [11], was applied as the primary classification in order to preserve direct comparability with the international sarcopenia-in-COPD literature, which almost uniformly uses the international cut-offs. The implications of this choice for an Indian cohort are addressed in the Discussion. Demographic and clinical data, including age, sex, smoking status, COPD severity, BMI, and sarcopenia status, were recorded for all participants.

Statistical analysis

Data were analysed using IBM SPSS Statistics for Windows, Version 27 (Released 2019; IBM Corp., Armonk, New York, United States). Categorical variables are reported as frequencies and percentages; continuous variables are reported as means. The Pearson chi-square (χ²) test was employed to evaluate associations between sarcopenia status and COPD severity, sex, BMI category, and age group. The assumption that no more than 20% of cells should have an expected count below five was checked for every contingency table; where this assumption was violated - as it was for the severity, BMI and age analyses - Fisher’s exact test was additionally computed and both results are reported. No continuity correction was applied to 2 × 2 tables. Prevalence estimates are accompanied by 95% Wilson score confidence intervals. Because the sample size and event counts were insufficient to support a stable multivariable model, no adjustment for potential confounders was undertaken; all reported associations are therefore unadjusted, and this is acknowledged as a limitation. All frequencies, percentages, chi-square statistics, exact tests and confidence intervals reported below were recomputed directly from the study dataset during revision and verified against the tabulated cell counts. A two-tailed p-value of <0.05 was considered statistically significant.

## Results

The study enrolled 100 patients, of whom 67 (67%) were male and 33 (33%) were female. The overall mean age was 56.69 years, which is younger than most comparable international COPD cohorts (Table 3).

**Table 3:** Age and sex distribution of study participants n = number of participants. Data are descriptive (frequency and percentage); no formal statistical comparison was performed for this table.

Age Group (years)	Male, n (%)	Female, n (%)	Total, n (%)
30–39	5 (55.6)	4 (44.4)	9 (9.0)
40–49	10 (58.8)	7 (41.2)	17 (17.0)
50–59	21 (70.0)	9 (30.0)	30 (30.0)
60–69	18 (66.7)	9 (33.3)	27 (27.0)
≥70	13 (76.5)	4 (23.5)	17 (17.0)
Total	67 (67.0)	33 (33.0)	100 (100.0)

As presented in Table 4, GOLD severity classification revealed that the majority of patients had advanced disease: GOLD 1 (mild) in three patients (3%), GOLD 2 (moderate) in 14 (14%), GOLD 3 (severe) in 40 (40%), and GOLD 4 (very severe) in 43 (43%).

**Table 4:** Distribution of COPD severity by GOLD classification and sex COPD: chronic obstructive pulmonary disease; GOLD: Global Initiative for Chronic Obstructive Lung Disease. Data are descriptive; no formal statistical comparison was performed for this table.

GOLD Grade	Male, n (%)	Female, n (%)	Total, n (%)
GOLD 1 – Mild	1 (33.3)	2 (66.7)	3 (3.0)
GOLD 2 – Moderate	8 (57.1)	6 (42.9)	14 (14.0)
GOLD 3 – Severe	26 (65.0)	14 (35.0)	40 (40.0)
GOLD 4 – Very Severe	32 (74.4)	11 (25.6)	43 (43.0)
Total	67 (67.0)	33 (33.0)	100 (100.0)

As presented in Table 5, gait speed assessment demonstrated that 32 of 67 male patients (47.8%) and nine of 33 female patients (27.3%) had impaired performance, with values at or below the 0.8 m/s threshold. Across the entire cohort, the mean gait speed in sarcopenic patients was 0.61 m/s (male patients) and 0.60 m/s (female patients), compared with 1.28 m/s and 1.24 m/s, respectively, in non-sarcopenic participants.

**Table 5:** Gait speed distribution by sex EWGSOP2: European Working Group on Sarcopenia in Older People 2019; 6MWT: six-minute walk test. Gait speed threshold ≤0.8 m/s per EWGSOP2 criteria. Data are descriptive. Three gait speeds originally recorded as 2.2-2.7 m/s were confirmed on rechecking to be transcription errors and corrected to 1.2, 1.4 and 0.9 m/s; all remained above the diagnostic threshold, so no classification or threshold count is affected.

Sex	Total	Gait Speed ≤0.8 m/s, n (%)	Gait Speed >0.8 m/s, n (%)
Male	67	32 (47.8)	35 (52.2)
Female	33	9 (27.3)	24 (72.7)

As presented in Table 6, low ASM/Ht² below the sex-specific EWGSOP2 threshold was identified in 27 of 67 male patients (40.3%) and 4 of 33 female patients (12.1%). The mean ASM/Ht² in sarcopenic male patients was 6.47 kg/m², compared with 10.92 kg/m² in non-sarcopenic men; the corresponding figures for women were 4.97 kg/m² and 9.84 kg/m², respectively.

**Table 6:** Muscle mass distribution by sex (ASM/Ht²) EWGSOP2 sex-specific appendicular skeletal muscle mass thresholds applied (men <7.0 kg/m²; women <5.5 kg/m²). Data are descriptive. The measured quantity was a skeletal muscle index obtained from a bipedal impedance device that does not report appendicular skeletal muscle mass directly; see Methods and Limitations. EWGSOP2: European Working Group on Sarcopenia in Older People 2019

Sex	Total	Below Threshold, n (%)	At or Above Threshold, n (%)
Male (threshold: <7.0 kg/m²)	67	27 (40.3)	40 (59.7)
Female (threshold: <5.5 kg/m²)	33	4 (12.1)	29 (87.9)

As presented in Table 7, handgrip strength below the sex-specific cut-off was recorded in 30 male patients (44.7%) and four female patients (12.1%). The mean grip strength among sarcopenic male patients was 21.87 kg versus 29.14 kg in non-sarcopenic men; for women, the respective values were 14.53 kg and 26.58 kg.

**Table 7:** Handgrip strength distribution by sex EWGSOP2: European Working Group on Sarcopenia in Older People 2. Sex-specific EWGSOP2 handgrip strength thresholds applied (men <27 kg; women <16 kg). Data are descriptive; no formal statistical comparison was performed for this table.

Sex	Total	Below Threshold, n (%)	At or Above Threshold, n (%)
Male (threshold: <27 kg)	67	30 (44.7)	37 (55.3)
Female (threshold: <16 kg)	33	4 (12.1)	29 (87.9)

As presented in Table 8, applying the full EWGSOP2 algorithm, sarcopenia was confirmed in 31 of 100 patients (31%; 95% CI 22.8-40.6). The prevalence was significantly higher in male patients (27/67, 40.3%; 95% CI 29.4-52.3) than in female patients (four of 33, 12.1%; 95% CI 4.8-27.3; p = 0.004).

**Table 8:** Prevalence of sarcopenia by sex Pearson chi-square (χ²) test; χ² = 8.21, df = 1, p = 0.004. Fisher’s exact test gave p = 0.005. All expected cell counts exceeded five, so the χ² assumption was satisfied. Statistical significance was predefined at p < 0.05.

Sex	Total	Sarcopenia Present, n (%)	Sarcopenia Absent, n (%)	p-value
Male	67	27 (40.3)	40 (59.7)	0.004
Female	33	4 (12.1)	29 (87.9)	
Overall	100	31 (31.0)	69 (69.0)	

All 31 cases satisfied both components of the EWGSOP2 confirmatory definition: every patient classified as sarcopenic had both a muscle index below the sex-specific threshold and handgrip strength below the sex-specific cut-off. No case was classified on the basis of low muscle mass together with impaired physical performance alone. A further three patients had low grip strength with a muscle index above the threshold and were therefore not classified as sarcopenic.

As presented in Table 9, the relationship between sarcopenia and COPD severity was examined after grouping patients into three severity tiers: mild (n = 3), moderate (n = 14), and severe-to-very-severe (n = 83). No patient in the mild category met sarcopenia criteria. Sarcopenia was present in five of 14 moderate COPD patients (35.7%) and in 26 of 83 severe-to-very-severe patients (31.3%). Although sarcopenia was numerically absent in mild disease and present in approximately one-third of patients in both the moderate and the severe-to-very-severe categories, this distribution did not reach statistical significance (χ² = 1.50, df = 2, p = 0.473). Because three of the six cells had expected counts below five, Fisher’s exact test was also computed and likewise showed no significant association (p = 0.632). Analysis across all four GOLD grades without collapsing gave the same result (sarcopenia in 0 of 3 mild, 5 of 14 moderate, 12 of 40 severe and 14 of 43 very severe patients; χ² = 1.56, df = 3, p = 0.668). The mild-disease stratum contained only three patients, so the observed 0% prevalence in that group carries essentially no statistical information, and the study was not powered to detect a severity gradient.

**Table 9:** Distribution of sarcopenia across COPD severity categories Pearson chi-square (χ²) test; χ² = 1.50, df = 2, p = 0.473. Fisher’s exact test: p = 0.632. Three of six cells had an expected count below five, so the exact test is the more reliable of the two. The difference across severity strata is not statistically significant. Analysis across all four GOLD grades gave χ² = 1.56, df = 3, p = 0.668. Statistical significance was predefined at p < 0.05. COPD: chronic obstructive pulmonary disease; GOLD: Global Initiative for Chronic Obstructive Lung Disease.

COPD Severity	Total	Sarcopenia Present, n (%)	Sarcopenia Absent, n (%)
Mild (GOLD 1)	3	0 (0.0)	3 (100.0)
Moderate (GOLD 2)	14	5 (35.7)	9 (64.3)
Severe + Very Severe (GOLD 3–4)	83	26 (31.3)	57 (68.7)
Total	100	31 (31.0)	69 (69.0)

As presented in Table 10, among the 31 patients with sarcopenia, 14 (45.2%) were underweight, 16 (51.6%) had normal BMI, none were overweight, and one (3.2%) was obese. Expressed by BMI stratum, sarcopenia affected 14 of 19 underweight patients (73.7%), 16 of 53 normal-weight patients (30.2%), none of the 23 overweight patients and one of the five obese patients (20.0%). The mean BMI was lower among sarcopenic than non-sarcopenic patients (19.58 vs. 24.04 kg/m²; Welch t-test p < 0.001), and the association between BMI category and sarcopenia status was highly significant (χ² = 26.82, df = 3, p < 0.001; Fisher’s exact p < 0.001).

**Table 10:** Sarcopenia stratified by BMI category Pearson chi-square (χ²) test; χ² = 26.82, df = 3, p < 0.001. Fisher’s exact test: p < 0.001. Two of eight cells had an expected count below five, so the exact test is reported alongside. Percentages in the “present” and “absent” columns are of the 31 sarcopenic and 69 non-sarcopenic patients respectively. This table has been corrected: the previously submitted version omitted 28 non-sarcopenic patients (17 normal-weight, 11 overweight) and reported χ² = 16.53 computed on the incomplete data.This is a cross-sectional association and does not imply that low BMI causes sarcopenia or the reverse. BMI: body mass index.

BMI Category	Sarcopenia Present, n (%)	Sarcopenia Absent, n (%)	Total, n (%)
Underweight (<18.5 kg/m²)	14 (45.2)	5 (7.2)	19 (19.0)
Normal (18.5–24.9 kg/m²)	16 (51.6)	37 (53.6)	53 (53.0)
Overweight (25.0–29.9 kg/m²)	0 (0.0)	23 (33.3)	23 (23.0)
Obese (≥30.0 kg/m²)	1 (3.2)	4 (5.8)	5 (5.0)
Total	31 (100.0)	69 (100.0)	100 (100.0)

As shown in Table 11, sarcopenia prevalence demonstrated a numerical increase with age, rising from 11.1% in the 30-39 year group to 41.2% in those aged 70 years or older; this trend did not achieve statistical significance (χ² = 3.08, df = 4, p = 0.545; Fisher’s exact p = 0.589).

**Table 11:** Sarcopenia prevalence across age groups Pearson chi-square (χ²) test; χ² = 3.08, df = 4, p = 0.545 (not statistically significant). One of ten cells had an expected count below five; Fisher’s exact test gave p = 0.589, confirming the absence of a significant association. Statistical significance was predefined at p < 0.05.

Age Group (years)	Total	Sarcopenia Present, n (%)
30–39	9	1 (11.1)
40–49	17	4 (23.5)
50–59	30	10 (33.3)
60–69	27	9 (33.3)
≥70	17	7 (41.2)
Total	100	31 (31.0)

Because the WHO international BMI cut-off points may not be appropriate for South Asian body composition, a post hoc sensitivity analysis repeated this classification (Table 12) using the Asian-specific action points recommended by the WHO Expert Consultation [11]. Under these thresholds the association remained highly significant (χ² = 26.42, df = 3, p < 0.001; Fisher’s exact p < 0.001), but the distribution changed materially: three sarcopenic patients with BMI values between 23.0 and 24.9 kg/m² (23.18, 23.63 and 23.87 kg/m²) were reclassified from normal weight to overweight, so sarcopenia was no longer absent above the normal-weight range, affecting 3 of 29 overweight (10.3%) and 1 of 15 obese patients (6.7%).

**Table 12:** Sensitivity analysis — sarcopenia stratified by Asian-specific BMI category Pearson chi-square (χ²) test; χ² = 26.42, df = 3, p < 0.001. Fisher’s exact test: p < 0.001. Asian-specific action points per the WHO Expert Consultation [11]: overweight ≥23.0 kg/m², obesity ≥27.5 kg/m². BMI: body mass index.

BMI Category (Asian-specific)	Sarcopenia Present, n (%)	Sarcopenia Absent, n (%)	Total, n (%)
Underweight (<18.5 kg/m²)	14 (45.2)	5 (7.2)	19 (19.0)
Normal (18.5–22.9 kg/m²)	13 (41.9)	24 (34.8)	37 (37.0)
Overweight (23.0–27.4 kg/m²)	3 (9.7)	26 (37.7)	29 (29.0)
Obese (≥27.5 kg/m²)	1 (3.2)	14 (20.3)	15 (15.0)
Total	31 (100.0)	69 (100.0)	100 (100.0)

## Discussion

This prospective cross-sectional observational study documents a sarcopenia prevalence of 31% (95% CI 22.8-40.6) among spirometry-confirmed COPD patients at a rural Indian tertiary hospital, using the EWGSOP2 diagnostic algorithm. The findings align with published pooled estimates from large international meta-analyses, which place overall sarcopenia prevalence in COPD between 21% and 29% [6,12], and the point estimate sits modestly above that range in a manner consistent with the advanced disease severity that predominated in this referral-based cohort.

Demographic profile and sarcopenia in younger COPD patients

The mean age of 56.69 years is substantially lower than the typical age in European and East Asian sarcopenia-in-COPD studies, where mean ages of 67-77 years are frequently reported [13-15]. This younger age at diagnosis reflects the earlier COPD onset characteristic of South Asian populations, driven by dual exposures to tobacco smoke and household biomass fuel combustion alongside occupational inhalants. The detection of sarcopenia in a relatively young cohort underscores the severity of disease-associated muscle loss in this population and calls attention to the limitations of extrapolating age-based sarcopenia thresholds from older Western or East Asian reference groups.

Nine patients (9%) were younger than 40 years, and one patient met criteria for sarcopenia. Sarcopenia in the fourth decade cannot be attributed to age-related muscle loss, and several aetiological factors may be relevant in this setting, although none was measured in this study and the following is therefore offered as context rather than as a finding. Early-onset COPD is a recognised entity with accelerated lung function decline and elevated exacerbation and mortality risk [16]. Non-smoking exposures dominate the aetiology of COPD in young Indian adults, including lifelong household biomass smoke exposure beginning in childhood, occupational dust, and childhood respiratory infection [17]. Prior pulmonary tuberculosis is a major cause of chronic airflow obstruction in young adults in India and itself produces wasting that may not fully recover [18]. Chronic undernutrition and low peak muscle mass may also mean that a smaller absolute loss of muscle crosses a fixed diagnostic threshold. The practical implication we draw is simply that young COPD patients should not be excluded from sarcopenia screening on the basis of age.

Sex differences

The sex difference in sarcopenia prevalence - male patients 40.3% versus female patients 12.1% (p = 0.004) - is consistent with global observations. Several explanations are commonly advanced for this pattern, none of which the present study is able to test. Oestrogen has been proposed to exert muscle-protective effects through modulation of inflammatory cytokine expression and anabolic signalling [19]. Differential smoking and occupational exposure between men and women in this setting is another possibility, but neither was quantified in this cohort, and the suggestion is therefore speculative rather than a finding of this study. He et al. and Yu et al. both report higher prevalence in men [12,20]. It should also be noted that sex-specific thresholds are applied within the EWGSOP2 algorithm, so the difference is not an artefact of a single cut-off; and the wide confidence interval around the female estimate (4.8-27.3%) reflects the small number of affected women and counsels caution.

Sarcopenia and COPD severity

Contrary to the direction suggested by much of the published literature, the present cohort did not show a statistically significant gradient of sarcopenia across GOLD severity strata (χ² = 1.50, df = 2, p = 0.473; Fisher’s exact p = 0.632). Sarcopenia was absent in the mild group, present in 35.7% of moderate disease, and present in 31.3% of severe-to-very-severe disease. Two features of the sample account for this null result more convincingly than any biological explanation. First, the distribution of severity was extremely skewed: 83 of 100 patients fell into the combined severe-to-very-severe category, while only three had mild disease. A three-patient stratum cannot support any inference, and with 83% of the cohort concentrated in a single tier there is very little variance in the exposure of interest to detect a gradient against. Second, the sample size was determined for prevalence estimation, not for stratified comparison, and is accordingly underpowered for this secondary objective. The apparent absence of an association should therefore be read as an absence of evidence rather than as evidence of absence.

Mechanistically, a severity gradient remains biologically plausible: progressive accumulation of systemic inflammatory mediators, increasing hypoxaemic burden, more pronounced physical inactivity, and greater cumulative corticosteroid exposure across advancing GOLD stages all provide a coherent rationale for muscle loss tracking with airflow obstruction [21,22]. The present null finding neither confirms nor refutes that rationale; the study simply lacks the severity distribution required to test it. It contrasts with data from Limpawattana et al. in a Southeast Asian cohort, where moderate and severe COPD conferred odds ratios of 19.2 and 13.4, respectively, for sarcopenia compared with mild disease [23], and with the meta-analytic findings of Zhou et al. showing that GOLD C and D disease more than doubles sarcopenia risk [24]. Those studies enrolled cohorts with a far broader spread of severity, including substantial numbers of GOLD 1 and 2 patients, which is precisely the contrast the present sample lacks. A multicentre study enrolling patients across the full GOLD spectrum, with prospective stratified sampling, is required to address this question properly.

Comparison with published prevalence estimates

The observed prevalence of 31% compares closely with the pooled estimate of 29% reported by Yu et al. across 15 studies involving 7,583 patients [12] and exceeds the 21.6% identified by Benz et al. in an earlier, smaller meta-analysis [6]. The higher estimates from certain studies, notably Costa et al. at 39.6% and Wu et al. at 52.5%, likely reflect differences in diagnostic criteria, the use of BODE index-stratified subgroups, or the higher mean age in those cohorts [15,25]. Studies applying Asian Working Group for Sarcopenia (AWGS) cut-off values, which use lower grip strength thresholds than EWGSOP2, tend to detect sarcopenia in a higher proportion of Asian patients, which may partially explain why the use of European criteria here yields an estimate towards the lower end of the range reported for regional settings.

Functional consequences

The functional deficits observed in sarcopenic patients were clinically substantial. Mean gait speeds of 0.61 m/s in sarcopenic male patients and 0.60 m/s in sarcopenic female patients both fall below the 0.8 m/s threshold associated with increased mortality, hospitalisation, and fall risk in older adults with chronic disease. Reductions in handgrip strength to 21.87 kg and 14.53 kg in sarcopenic male patients and female patients, respectively, against cut-offs of 27 kg and 16 kg, reflect a clinically relevant degree of muscular weakness. These measurements translate directly into reduced capacity for pulmonary rehabilitation participation, greater dependence in activities of daily living, and higher susceptibility to acute exacerbation-related functional decline [26]. Because these functional variables form part of the diagnostic definition of sarcopenia, the differences between sarcopenic and non-sarcopenic patients are partly definitional and should not be interpreted as independent findings.

Sarcopenia, BMI, and the limits of body weight as a surrogate

The mean BMI of sarcopenic patients was 19.58 kg/m², consistent with a nutritionally depleted phenotype, and underweight COPD patients are recognised to have worse prognosis independent of airflow limitation [27]. The present study demonstrates an association between lower BMI and sarcopenia; its cross-sectional design cannot establish that low BMI causes sarcopenia, that muscle loss drives the fall in BMI, or that a third factor such as disease severity or nutritional intake underlies both. The direction of any such relationship is untestable in these data.

Of particular interest is the distribution of sarcopenia across BMI strata. Sixteen of the 31 sarcopenic patients (51.6%) had a BMI within the normal range and a further 14 (45.2%) were underweight; no sarcopenic patient was overweight and only one was obese. Read the other way, sarcopenia affected 73.7% of underweight patients and 30.2% of normal-weight patients, but none of the 23 overweight patients and only one of the five obese patients. Three explanations account for this pattern. First, BMI is a measure of total body mass and cannot distinguish fat mass from fat-free mass; a patient may lose several kilograms of appendicular lean tissue while gaining or maintaining fat mass, leaving BMI unchanged. This is the well-documented phenomenon of sarcopenia with preserved body weight, and it is why EWGSOP2 requires direct measurement of muscle mass rather than any weight-based surrogate [8]. Tsekoura et al. and Costa et al. both documented sarcopenia in individuals with normal or elevated BMI, emphasising that normal body weight does not exclude clinically significant muscle loss [13,25]. Second, at the upper end of the BMI range, sarcopenia does not disappear but changes phenotype: sarcopenic obesity, for which ESPEN and EASO published formal diagnostic criteria in 2022, describes the coexistence of excess adiposity with low muscle mass and function, and is systematically underdetected by muscle mass indexed to height squared, because height-indexed indices such as ASM/Ht² are positively biased by the greater absolute lean mass that accompanies higher body weight [28]. Indexing to weight or to BMI, or applying the ESPEN/EASO algorithm, would be expected to identify additional cases among the 23 overweight and five obese patients in this cohort, of whom only one was classified as sarcopenic under the height-indexed criterion used here. Third, adipose tissue in COPD is not metabolically inert, and higher fat mass has been associated in some cohorts with better short-term survival, the so-called obesity paradox; whether this reflects preserved nutritional reserve, reverse causation from disease-driven weight loss, or residual confounding remains unresolved [27]. The practical implication is that the absence of sarcopenia among overweight patients in this study should be interpreted as a limitation of the height-indexed diagnostic criterion rather than as evidence that a higher BMI is protective. Applying the WHO international rather than the Asian-specific BMI action points [10,11] compounds this, since a proportion of patients classified here as normal weight would be classified as overweight under Asian cut-offs. Age was not significantly associated with sarcopenia in this cohort, possibly because of the relatively narrow age distribution and limited sample size.

Clinical implications

The findings reinforce the clinical case for incorporating structured sarcopenia screening into routine COPD assessment in India. The three components of the EWGSOP2 algorithm, BIA-based muscle mass measurement, handheld dynamometry, and gait speed testing, are affordable, portable, and feasible in resource-limited settings. Identification of sarcopenia should trigger a multidisciplinary response encompassing supervised pulmonary rehabilitation with a progressive resistance training component, individualised nutritional assessment with protein intake optimisation (targeting ≥1.0-1.2 g/kg/day), and correction of micronutrient deficiencies, particularly vitamin D [29]. Data indicate that sarcopenia prevalence can decrease substantially following structured pulmonary rehabilitation; Jones et al. documented a reduction from approximately 22% to 7% after a rehabilitation programme, highlighting the reversibility of at least a portion of COPD-related muscle loss [30].

Strengths

This study has several strengths. It addresses a clinically important question for which Indian data are almost entirely absent, and it does so in a rural tertiary care population that is under-represented in the sarcopenia literature. Enrolment was prospective and consecutive rather than retrospective or convenience-based, which limits selection bias at the point of recruitment. The full three-component EWGSOP2 algorithm was applied, rather than muscle mass alone, so cases meet an internationally recognised operational definition and are directly comparable with published cohorts; many studies in this field substitute a single surrogate measure. The same instruments were used for all participants throughout the study period, limiting inter-instrument variability. Exclusion of patients with major comorbidities known to affect muscle independently of COPD, while it narrows generalisability, allows the observed sarcopenia burden to be attributed to COPD with greater confidence than in unselected cohorts. Finally, the analysis reports exact tests alongside chi-square statistics and confidence intervals around prevalence estimates, and does not overstate associations that the sample cannot support.

Limitations

The most substantial limitation concerns the assessment of muscle mass. EWGSOP2 notes that bioelectrical impedance does not measure muscle mass directly but derives an estimate through a conversion equation calibrated against dual-energy X-ray absorptiometry in a specific population and advises that raw device measures be combined with a cross-validated equation for standardisation [8]. The device used here is a bipedal, single-frequency, home-use body composition monitor that does not report appendicular skeletal muscle mass, performs no segmental analysis, and does not display raw resistance or reactance, so that a standardisation approach was not available. The muscle index recorded was compared against EWGSOP2 thresholds derived for appendicular skeletal muscle mass, and no conversion equation is documented for it. The manufacturer’s stated accuracy for its muscle output is ±3.5%; measurements may be unreliable in the presence of oedema, and the estimating algorithms are proprietary. The prevalence estimate reported here should therefore be interpreted as applying to this index rather than as directly comparable with studies using dual-energy X-ray absorptiometry or research-grade multi-frequency impedance. Muscle mass was not measured by DXA, the reference standard.

Gait speed was obtained from a six-minute walk rather than the four-metre usual-pace walk specified by EWGSOP2, and the two are not equivalent. The arithmetic used to derive it was not documented and the walk distances were not retained. Three of the 100 recorded values (2.2-2.7 m/s) are not physiologically plausible. On re-checking against the source records, they were confirmed as transcription errors and corrected to 1.2, 1.4 and 0.9 m/s. The correction lowers the mean gait speed of non-sarcopenic patients from 1.28 to 1.22 m/s in men and from 1.24 to 1.18 m/s in women. All three belonged to non-sarcopenic patients and remained above the 0.8 m/s threshold after correction, so the threshold counts in Table 5 and every reported test statistic are unaffected.

The single-centre design and modest sample size limit external generalisability. Referral bias is substantial: 83% of participants had severe or very severe airflow obstruction, whereas population-based COPD cohorts are dominated by GOLD 1 and 2 disease, so the prevalence estimate of 31% should be read as applying to a severe-disease referral population, and the near-absence of mild disease is the principal reason the severity analysis was uninformative. The exclusion of patients with major comorbidities narrows applicability to a real-world population in which multimorbidity is the norm. The cross-sectional design precludes any inference about causal directionality; where mechanisms are discussed above, they are drawn from the published literature and are not inferences from these data.

No adjustment was made for potential confounders. Smoking history was elicited at enrolment but was not quantified in pack-years or entered into any analysis, and cumulative corticosteroid exposure, dietary protein intake, habitual physical activity, socioeconomic status, degree of hypoxaemia, and inflammatory biomarkers were not measured at all. All reported associations are therefore unadjusted univariate comparisons, and the explanations offered in the Discussion for the observed sex difference are hypotheses drawn from the literature rather than results of this study. Handgrip strength was measured with a strain-gauge rather than a Jamar hydraulic dynamometer, from which the EWGSOP2 thresholds were principally derived, so the proportion classified as weak may differ modestly. Several further procedural details were not recorded and cannot be reconstructed: participant positioning and rest interval during dynamometry, the length of the walking course, whether impedance measurements were standardised for fasting state, hydration and time of day, the order of the three assessments, and the number of patients screened and excluded. The analyses of sex, age and BMI were not pre-specified and are exploratory; no correction for multiple comparisons was applied. Finally, sarcopenia severity was not graded, and no follow-up data are available.

## Conclusions

Sarcopenia is a prevalent comorbidity in COPD, affecting a substantial proportion of patients in this tertiary care cohort. It was significantly more frequent among male patients and was concentrated among underweight and normal-weight individuals. Prevalence was numerically higher in moderate and advanced disease than in mild disease, but no statistically significant gradient across GOLD severity strata could be demonstrated; this reflects the narrow severity distribution of the sample rather than evidence against a severity relationship.

All associations reported here are cross-sectional and non-causal, and the assessment of muscle mass departed from the reference method in ways set out in the Limitations. The functional impairments observed in sarcopenic patients nonetheless reinforce the clinical case for early detection. Routine integration of muscle mass and functional assessment into COPD management protocols is feasible in resource-limited settings, and multidisciplinary intervention combining structured exercise rehabilitation with nutritional support offers the most evidence-based pathway to attenuate sarcopenia in this population. Future prospective, multicentre investigations enrolling patients across the full spectrum of GOLD severity, using reference-standard body composition measurement and Asian-specific diagnostic criteria, are needed to clarify these relationships.
